# Protein Phosphatase Methyl-Esterase PME-1 Protects Protein Phosphatase 2A from Ubiquitin/Proteasome Degradation

**DOI:** 10.1371/journal.pone.0145226

**Published:** 2015-12-17

**Authors:** Ryotaro Yabe, Akane Miura, Tatsuya Usui, Ingrid Mudrak, Egon Ogris, Takashi Ohama, Koichi Sato

**Affiliations:** 1 Laboratory of Veterinary Pharmacology, Joint Faculty of Veterinary Medicine, Yamaguchi University, Yamaguchi, 753–8515, Japan; 2 Laboratory of Veterinary Toxicology, Joint Faculty of Veterinary Medicine, Yamaguchi University, Yamaguchi, 753–8515, Japan; 3 Department of Medical Biochemistry, Max F. Perutz Laboratories, Medical University of Vienna, Vienna, 1030, Austria; Faculty of Medicine, BELGIUM

## Abstract

Protein phosphatase 2A (PP2A) is a conserved essential enzyme that is implicated as a tumor suppressor based on its central role in phosphorylation-dependent signaling pathways. Protein phosphatase methyl esterase (PME-1) catalyzes specifically the demethylation of the C-terminal Leu309 residue of PP2A catalytic subunit (PP2Ac). It has been shown that PME-1 affects the activity of PP2A by demethylating PP2Ac, but also by directly binding to the phosphatase active site, suggesting loss of PME-1 in cells would enhance PP2A activity. However, here we show that PME-1 knockout mouse embryonic fibroblasts (MEFs) exhibit lower PP2A activity than wild type MEFs. Loss of PME-1 enhanced poly-ubiquitination of PP2Ac and shortened the half-life of PP2Ac protein resulting in reduced PP2Ac levels. Chemical inhibition of PME-1 and rescue experiments with wild type and mutated PME-1 revealed methyl-esterase activity was necessary to maintain PP2Ac protein levels. Our data demonstrate that PME-1 methyl-esterase activity protects PP2Ac from ubiquitin/proteasome degradation.

## Introduction

Protein phosphorylation is a regulatory mechanism for most cellular processes that requires coordination of protein kinases and protein phosphatases. Over 98% of protein phosphorylation occurs on serine and threonine residues, catalyzed by about 400 Ser/Thr kinases [1, 2]. On the other hand, dephosphorylation of Ser/Thr involves a much smaller number of phosphatases (about 40) distributed into three different enzyme superfamilies [3]. Protein phosphatases of the PPP superfamily account for most of the Ser/Thr phosphatase activity and assemble into hundreds of different multi-subunit holoenzymes [4]. Among these PPP phosphatases PP2A is highly conserved across eukaryotes from yeasts to human and involved in control of numerous signaling pathways, including cell cycle, apoptosis, and development [5]. Accumulating evidence has revealed that PP2A acts as a tumor suppressor and its inhibition can play a critical role in mammalian cell transformation [6, 7].

PP2A forms heterotrimers, each comprised of a catalytic subunit (C, or PP2Ac), a scaffolding subunit (A, or PP2A-A) and one regulatory B subunit from 4 different families of genes [5]. Regulatory B subunits control PP2A specificity by targeting PP2Ac to substrates. Mammalian cells also contain a pool of the AC core dimer [8]. Generation of active PP2A is tightly coupled to holoenzyme assembly [9]. Reversible carboxyl-terminus methyl esterification of PP2Ac Leu309 seems to be part of the mechanism for proper biogenesis of PP2A holoenzymes. The methylation of PP2Ac is catalyzed by adenosylmethionine-dependent leucine carboxyl methyltransferase (LCMT-1, also known as PMT-1) [10]. Methylation of PP2Ac stabilizes interactions with the other subunits, promoting formation of heterotrimers [11, 12].

Demethylation of PP2Ac is regulated by a specific methyl-esterase PME-1 [13]. Structural studies revealed that in addition to its role as PP2Ac methyl-esterase, PME-1 blocks enzyme activity by directly binding to the active site of PP2Ac [14]. As with other PP2A inhibitors such as SET and CIP2A, increased PME-1 expression correlates with disease progression in human cancer [15–18]. Being both a PP2Ac methyl-esterase and a direct inhibitor, levels of PME-1 could play a crucial role in determining levels of PP2A activity. However, the effects of PME-1 deficiency on PP2A have not been fully investigated. Here we reveal that knockout of PME-1 correlates with a decrease in PP2Ac protein levels, and PME-1 methyl-esterase activity protects PP2Ac from ubiquitin/proteasome degradation.

## Material and Methods

### Cell Culture

Mice were anesthetized with diethyl ether and euthanized by exsanguination. Mouse embryonic fibroblasts (MEFs) were isolated from embryos (ED12.5–14.5) of wild type and PME-1 KO mice [19]. All cells were grown in DMEM containing 10% FBS and 1x anti-biotic/anti-mycotic (Life Technologies, Carlsbad, CA, USA). All experiments and animal care procedures in this study were performed according to the Guide to Animal Use and Care of the Yamaguchi University and were approved by the ethics committee. HT29, 293, 293T, and A549 cells were grown in DMEM containing 10% FBS and 1x anti-biotic/anti-mycotic.

### Antibodies

Antibodies were obtained from the indicated supplier: anti-PP2A A subunit (Santa Cruz Biotech, CA, USA, sc-6112), anti-PP4c (Bethyl, TX, USA), anti-phospho ERK1/2, anti-phospho Thr308 Akt, anti-total ERK1/2, anti-total Akt (Cell Signaling, MA, USA), anti-FLAG tag (Sigma, MO, USA), anti-ubiquitin (Life Sensors, PA, USA), anti-PME-1 (LifeSpan BioScience, WA, USA), anti-demethyl PP2Ac (Merck Millipore, MA, USA, 05–577), anti-total PP2Ac (Millipore, 07–324), anti-tubulin alpha (Thermo Scientific, MA, USA), p97/VCP (GeneTex, CA, USA). Anti-PP6c was generated as previously described [20].

### Plasmid Contraction and Lentivirus Production

Human PME-1 wild type (WT) and S156A of pET-45b plasmids [21] were subcloned into BamHI/NotI sites of pLVSIN-EF1α-IRES-ZsGreen1 vector (Takara Bio, Shiga, Japan). Human PP2Acα WT and K41R were PCR amplified from pKMyc-PP2Acα plasmids [22] and pBabe HA-PP2AcαK41R and subcloned into EcoRI/BamHI site of pLVTetOn vector. FLAG tagged human PP2A B55α and B56α were PCR amplified from human liver cDNA and subcloned into pLVSIN-EF1α-IRES-ZsGreen1 vector. shRNA complementary DNA strands (19mer sequences: shNontarget (shNT), 5’-CAACAAGATGAGAGCACCA-3’, shPME-1, 5’GGCGATACATCTGAGTTCA-3’) with flanking sequences were annealed and ligated into the MluI/ClaI sites of pLV-mC [23] To produce lentiviruses, 3 *μ*g of pLVSIN, 2.3 *μ*g of a packaging plasmid (psPAX2) and 1.3 *μ*g of a coat-protein plasmid expressing vesicular stomatitis virus G protein (pMD2.G) were transfected into Lenti-X 293T cells cultured in 60-mm dishes using PEI Max (Polysciences, PA, USA) according to the manufacturer’s instruction. Viral supernatants were collected after 48 hr, and after filtering (0.22 *μ*m), were added to cells for 8 hr.

### Immunoblotting

The cells were lysed in a buffer containing 50 mM Tris-HCl (pH 8.0), 5 mM EDTA, 5 mM EGTA, 1% Triton X100, 1 mM Na_3_VO_4_, 20 mM sodium pyrophosphate, and Roche Complete protease inhibitor mixture. For measuring PP2Ac methylation level, 1 μM of ABL127 (PME-1 inhibitor) was added in a lysate buffer. The proteins were separated by SDS-PAGE and transferred onto PVDF membrane (Bio-Rad) or nitrocellulose membrane (Wako, Osaka, Japan). The membranes were blocked with 0.5% skim milk and treated with antibodies described and detected using ECL Pro (PerkinElmer, Freiburg, Germany). Bands of proteins were visualized using LAS-3000 (Fujifilm, Tokyo, Japan). Band densities were quantified using ImageJ densitometry analysis software (National Institutes of Health, Bethesda, MD, USA).

### Immunoprecipitation

Cells expressing FLAG-tagged PP2A or PME-1 were lysed in buffer consisting of 50 mM Tris-HCl (pH 8.0), 150 mM NaCl, 5 mM EDTA, 5 mM EGTA, 1% Triton X100, 1 mM Na_3_VO_4_, 20 mM sodium pyrophosphate, and Roche Complete protease inhibitor, sonicated 4 times for 5 sec each, then the lysates centrifuged at 13,000 g for 10 min. Supernatants (cell extracts) were incubated with FLAG M2 beads (Life Technologies) at 4°C for 2 h. Beads were washed three times with lysis buffer. Bound proteins were eluted with 3xFLAG peptide (Sigma) and subjected to immunoblotting. For endogenous PP2Ac immunoprecipitation, anti-PP2Ac (Millipore, 05–421) coupled with Dynabeads protein G (Life Technologies) was used.

### Phosphatase Activity Assay

PP2A activity in whole cell lysate was analyzed by using Ser/Thr Phosphatase Assay Kit 1 (Millipore) with threonine phosphopeptide (K-R-pT-I-R-R) as a substrate. PP2A phosphatase activity was calculated by subtracting activity in the presence of 10 nM okadaic acid. Immunoprecipitation based PP2A specific activity was analyzed by using Active PP2A DuoSet IC kit (R&D systems, MN, USA) according to the manufacturer’s instruction.

### Realtime qRT-PCR

Total RNA was extracted from cells by using TRIzol Reagents (Life Technologies). 0.5 μg of RNA was reverse transcribed in a final incubation volume of 10 μl using QuantiTect Reverse Transcription Kit (Qiagen, Hilden, Germany). The resulting cDNA was subjected to quantitative PCR using QuantiTect SYBR Green I Kit (Qiagen) and StepOne Plus (Applied Biosystems, CA, USA). The sequences of primers: PP2Acα forward CCTCTGCGAGAAGGCTAAAG, reverse GCCCATGTACATCTCCACAC, PP2Acβ forward CGAGTGTAAGCAGCTGAACG, reverse ATTTCCTTAGCCTTCTCGCA, GAPDH forward AGGTTGTCTCCTCTGACTTC, reverse TACCAGGAAATGAGCTTGAC. PP2Ac expression was normalized to GAPDH. The relative quantitative value for PP2Ac compared with GAPDH was expressed as comparative Ct (2^–(ΔCt-Cc)^) method.

### Cell Proliferation Assay

For cell proliferation assay for MEFs, 1 x 10^4^ cells were seeded on 96 well plates and cultured for up to 4 days. Cell Counting Kit-8 (Dojindo, Kumamoto, Japan) was used according to the manufacturer’s protocol.

### Statistical Analysis

The results are expressed as the means ± S.E. Comparisons between the groups were performed by one-way analysis of variance, followed by Student-Newman-Keuls test. For all of the analyses, a probability value of *p*<0.05 was considered to indicate statistical significance.

## Results

### PME-1 Knockout Reduces Whole Cell PP2A Activity

Targeted disruption of the *PME-1* gene causes perinatal lethality in mice [19]. To study the functional relationship between PME-1 and PP2A, we isolated mouse embryonic fibroblasts (MEFs) from ED12.5–14.5 embryos of wild type (WT) and PME-1 knockout (KO) littermates. The microscopic appearance of PME-1 KO MEFs was very similar to WT MEFs. We used these primary (non-immortalized) MEFs in this report.

**Fig 1:**
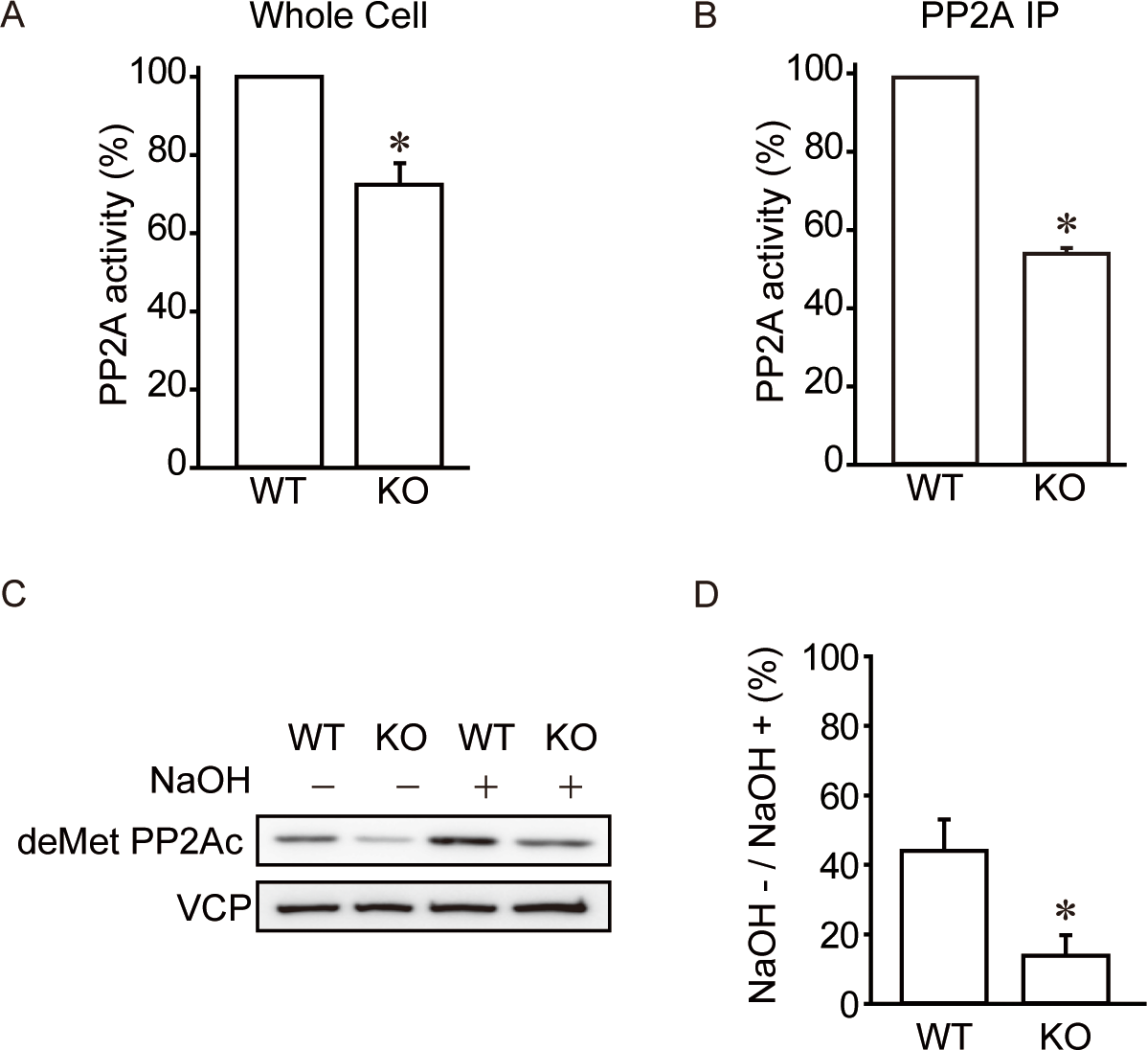
PME-1 knockout reduces whole cell PP2A activity. (A) Loss of PME-1 suppresses whole cell PP2A activity. PP2A phosphatase activity was analyzed with phospho-peptides as a substrate and calculated as difference between samples ± 10 nM okadaic acid. N = 4. *: *P*<0.05 vs. WT. (B) Loss of PME-1 suppresses PP2A specific activity. PP2A specific phosphatase activity was analyzed with immunoprecipitation based PP2A activity assay. N = 4. *: *P*<0.05 vs. WT. (C-D) Loss of PME-1 enhances PP2Ac methylation. Cell lysates were treated with or without NaOH. The ratio of demethylated PP2Ac was determined by immunoblotting for demethylated PP2Ac (deMet PP2Ac). Representative picture (C) and quantitative data (D) from 3 independent experiments are shown. *: *P*<0.05 vs. WT.

Because PME-1 can inhibit PP2A by direct association to the active site, we expected an increase in PP2A activity in PME-1 KO MEFs relative to WT MEFs. We assayed phosphatase activity in whole cell extracts of WT and PME-1 KO MEFs with a relatively specific PP2A phosphopeptide (K-R-pT-I-R-R) substrate, and calculated the difference between ± 10 nM okadaic acid as the PP2A activity (Fig 1A). Unexpectedly, PP2A activity in PME-1 KO MEFs was only about 70% of the activity in WT MEFs. To confirm this, we also performed an immunoprecipitation based PP2A-specific activity assay, and found about 50% decrease in PP2A activity in PME-1 KO MEFs (Fig 1B).

We next analyzed the relative fraction of methylated and demethylated PP2A by immunoblotting analysis using an antibody specific for demethylated Leu309 in PP2Ac, before and after reaction with strong base (0.1 M NaOH) to hydrolyse the methylester bond (Fig 1C and 1D). These studies estimated that ~50% and >90% of PP2Ac is methylated in PME-1 WT and KO MEFs, respectively. These results are in close agreement to the previous analyses with embryonic brain tissues [19].

### PME-1 Knockout Reduces PP2A Protein Levels

**Fig 2:**
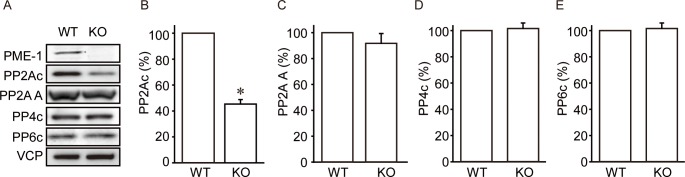
PME-1 knockout reduces PP2A protein levels. (A-E) Loss of PME-1 affects specifically PP2Ac protein levels. Levels of proteins in wild type (WT) and PME-1 KO (KO) MEFs were determined by immunoblotting and representative images (A) and quantitative data for PP2Ac (B), PP2A A (C), PP4c (D), and PP6c (E) from 3 independent experiments are shown. *: *P*<0.05 vs. WT.

How was PP2A activity decreased in KO MEFs? We realized that staining intensity for PP2Ac in NaOH treated cell lysates was quite different between WT and KO MEFs using antibodies directed to the C-terminus (Fig 1C). These immunoblots show the total PP2Ac levels, because all of PP2Ac was demethylated by strong base in these samples. To confirm this, we detected PP2Ac protein levels by a different anti-PP2Ac antibody. We used a polyclonal antibody against a peptide corresponding to amino acids 290–304 (DPAPRRGEPHVTRRT) of human PP2A (Millipore #07–324), which is not affected by the methylation status of Leu309 in PP2Ac. We confirmed that PME-1 knockout cells had significantly reduced levels of PP2Ac by about 60% with only minor changes for A subunit levels (Fig 2A–2C). Levels of PP4c and PP6c, other closely related type 2A protein phosphatases, were not affected by PME-1 knockout (Fig 2A, 2D and 2E). We concluded that loss of PME-1 specifically reduced PP2Ac protein levels without compensation by the most closely related PPP phosphatases, and this resulted in the lower PP2Ac activity in PME-1 KO MEFs.

We also investigated whether the effects of PME-1 loss on PP2Ac protein level is specific for MEFs. PME-1 expression was suppressed by shRNA in A549, 293, and HT29 cells (S1 Fig). PP2Ac protein level was decreased by PME-1 knockdown in A549 cells, but not in 293 and HT29, indicating that the effect of PME-1 loss on PP2Ac protein levels is dependent on cell types.

### PME-1 Protects PP2Ac from Ubiquitin/Proteasome Degradation

**Fig 3:**
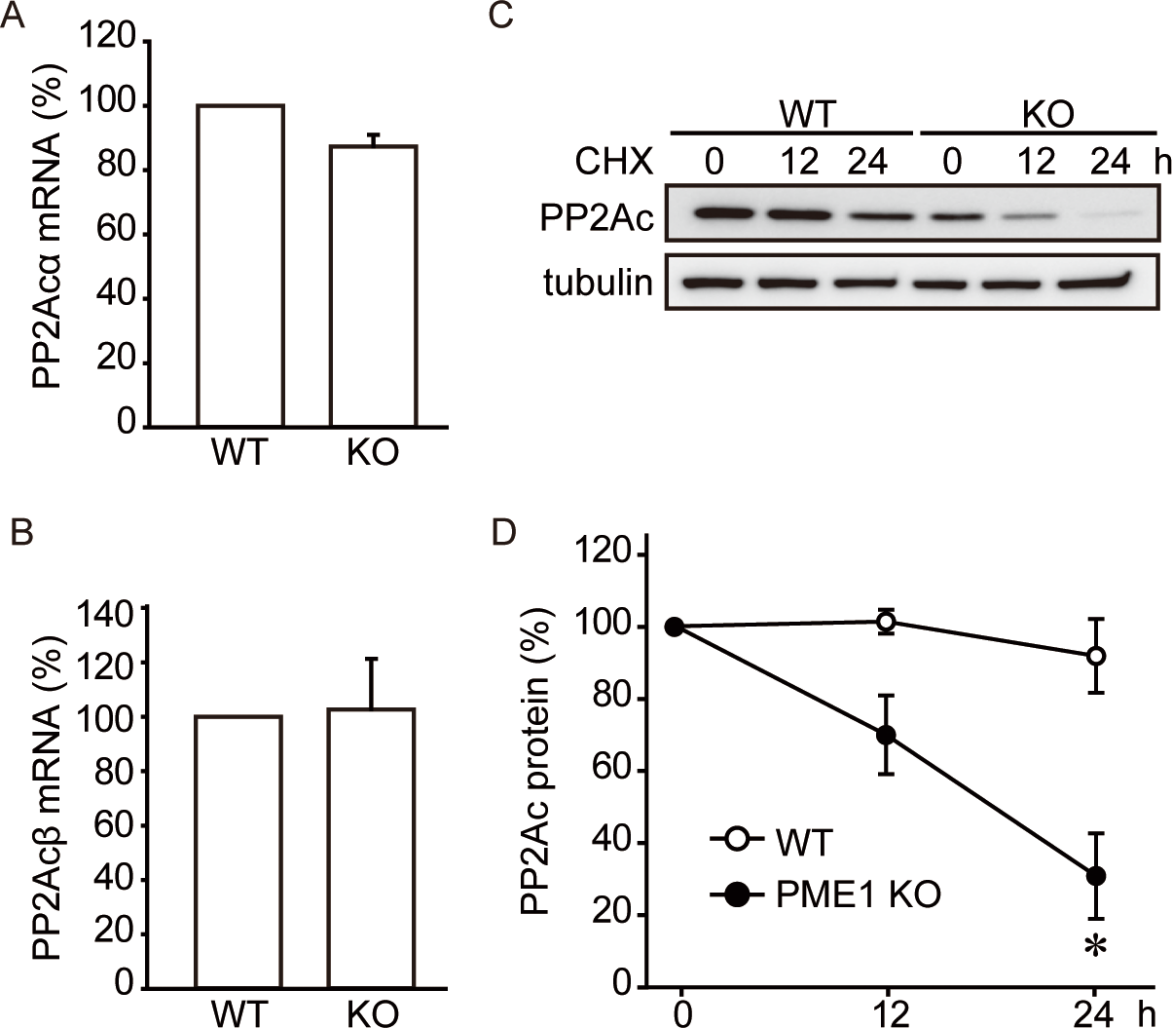
PME-1 protects PP2Ac from protein degradation. (A-B) Loss of PME-1 does not affect PP2Ac mRNA expression. mRNA levels of PP2Ac α (A) and β (B) isoforms were analyzed by real-time qPCR. Quantitative data from 2 independent experiments performed in duplicate are shown. (C-D) PME-1 protects PP2Ac from protein degradation. PP2Ac degradation was analyzed by cycloheximide chase assay. Representative images (C) and quantitative data for PP2Ac protein level (D) from 5 independent experiments are shown. *: *P*<0.05 vs. WT.

To determine how PME-1 knockout caused reduced levels of PP2Ac, we examined PP2Ac mRNA expression in WT and PME-1 KO MEFs by real time RT-qPCR. The mRNA levels for both isoforms of PP2Ac (PP2Acα and PP2Acβ) were not significantly different between WT and KO cells (Fig 3A and 3B) pointing to post-transcriptional mechanism(s).

Next, we examined PP2Ac protein half-life by blocking *de novo* synthesis with cycloheximide (CHX) and anti-total PP2Ac antibody (Millipore #07–324) (Fig 3C and 3D). Cells were treated with CHX and lysed after 0, 12 and 24 hr. In WT MEFs PP2Ac protein levels only slightly decreased by CHX treatment suggesting PP2Ac has a relatively long half-life. In contrast, PP2Ac levels in PME-1 KO MEFs rapidly decreased after addition of cycloheximide, with only about 35% of the protein remaining after 24 hr. These data indicate PME-1 affects PP2Ac stability, and our hypothesis was that PME-1 protects PP2Ac from protein degradation.

**Fig 4:**
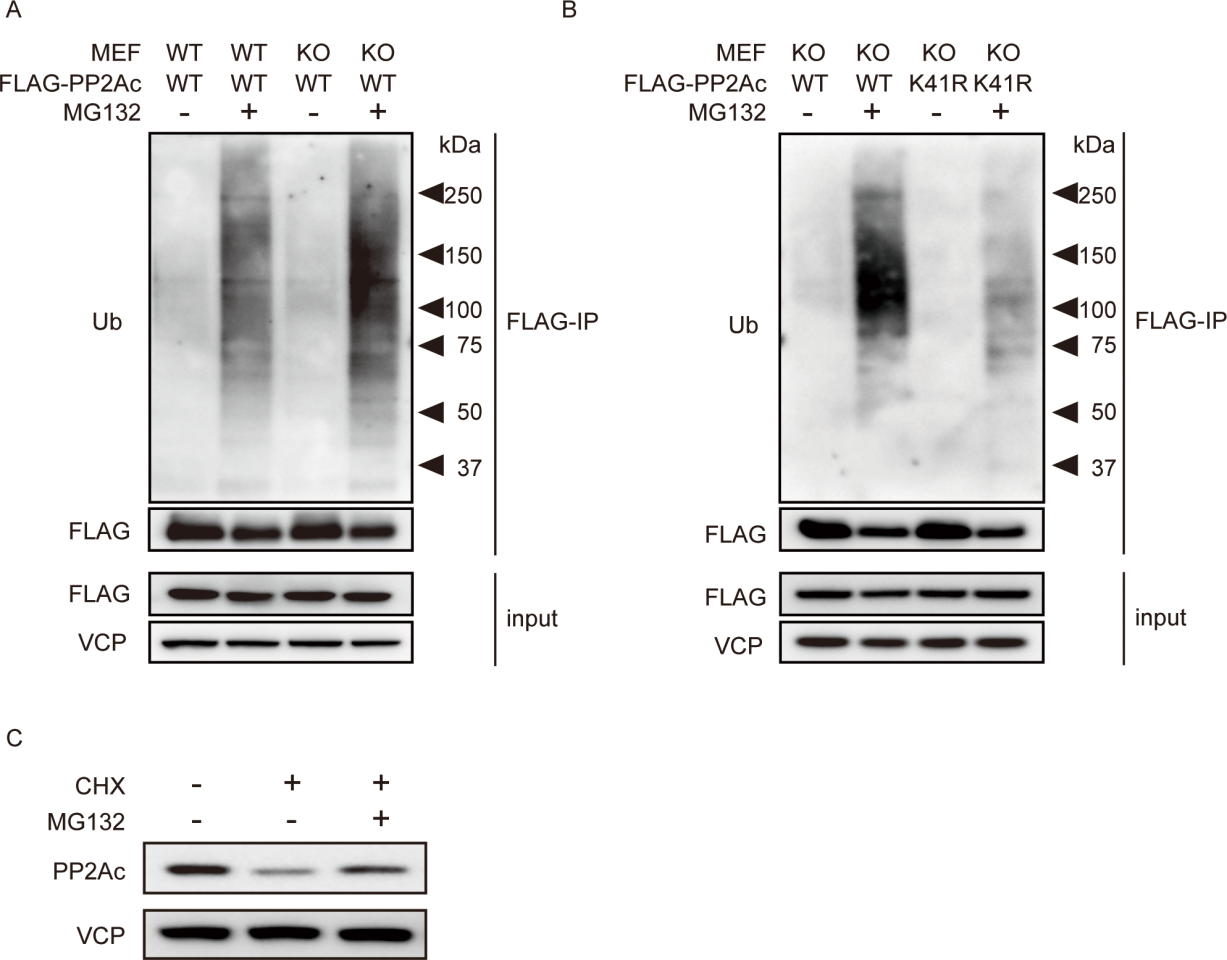
PME-1 knockout enhances PP2Ac poly-ubiquitination. (A) Loss of PME-1 enhances PP2Ac ubiquitination. FLAG-PP2Ac was transiently expressed by TetOn system in WT and PME-1 KO MEFs, and cells were treated with or without MG132 (10 μM) for 16 h. FLAG-PP2Ac was immunoprecipitated with FLAG-M2 beads, and ubiquitinated PP2Ac was detected by immunoblotting. Representative images from 2 independent experiments were shown. (B) K41R mutation of PP2Ac blocks PP2Ac ubiquitination. FLAG-PP2Ac WT and K41R mutants were transiently expressed by TetOn system in PME-1 KO MEFs, and cells were treated with or without MG132 (10 μM) for 16 h. FLAG-PP2Ac was immunoprecipitated with FLAG-M2 beads, and ubiquitinated PP2Ac was detected by immunoblotting. Representative images from 2 independent experiments were shown. (C) Proteasome inhibitor blocks PP2Ac degradation. PME-1 KO MEFs were treated with 25 μM cycloheximide with or without 10 μM MG132. Endogenous PP2Ac protein level was detected by immunoblotting. Representative images from 3 independent experiments were shown.

Accumulating evidence shows that levels of PP2Ac can be regulated by the poly-ubiquitin/proteasome system. To test whether PP2Ac poly-ubiquitination was affected by loss of PME-1, FLAG tagged PP2Ac was expressed by a TetOn system in WT and KO MEFs. After treatment with doxycycline (dox) for 48 hr, cells were treated with or without proteasome inhibitor MG132 for 16 hr and FLAG-PP2Ac was immunoprecipitated with FLAG M2 beads. We observed higher levels of poly-ubiquitinated FLAG-PP2Ac in PME-1 KO MEFs compared to WT MEFs (Fig 4A). These poly-ubiquitinated forms were significantly reduced by K41R mutation of PP2Ac, confirming this as the primary site of poly-ubiquitination of PP2Ac (Fig 4B). Moreover, proteasome inhibitor MG132 blocked CHX induced degradation of PP2Ac in PME-1 KO MEFs (Fig 4C). Together, these data indicate that loss of PME-1 leads to enhanced PP2Ac poly-ubiquitination and proteasomal degradation. To note, we observed higher FLAG-PP2Ac protein levels in WT MEFs compared with KO MEFs treated with dox for 24 hr (S2A Fig). FLAG-PP2Ac K41R protein level was higher than WT FLAG-PP2Ac in KO MEFs after 24 hr treatment with dox (S2B Fig). However, these differences were not observed after 64 (48 +16) hr treatments with dox (Fig 4A and 4B; also see discussion).

### Methyl-Esterase Activity of PME-1 Is Required to Maintain Levels of PP2Ac

**Fig 5:**
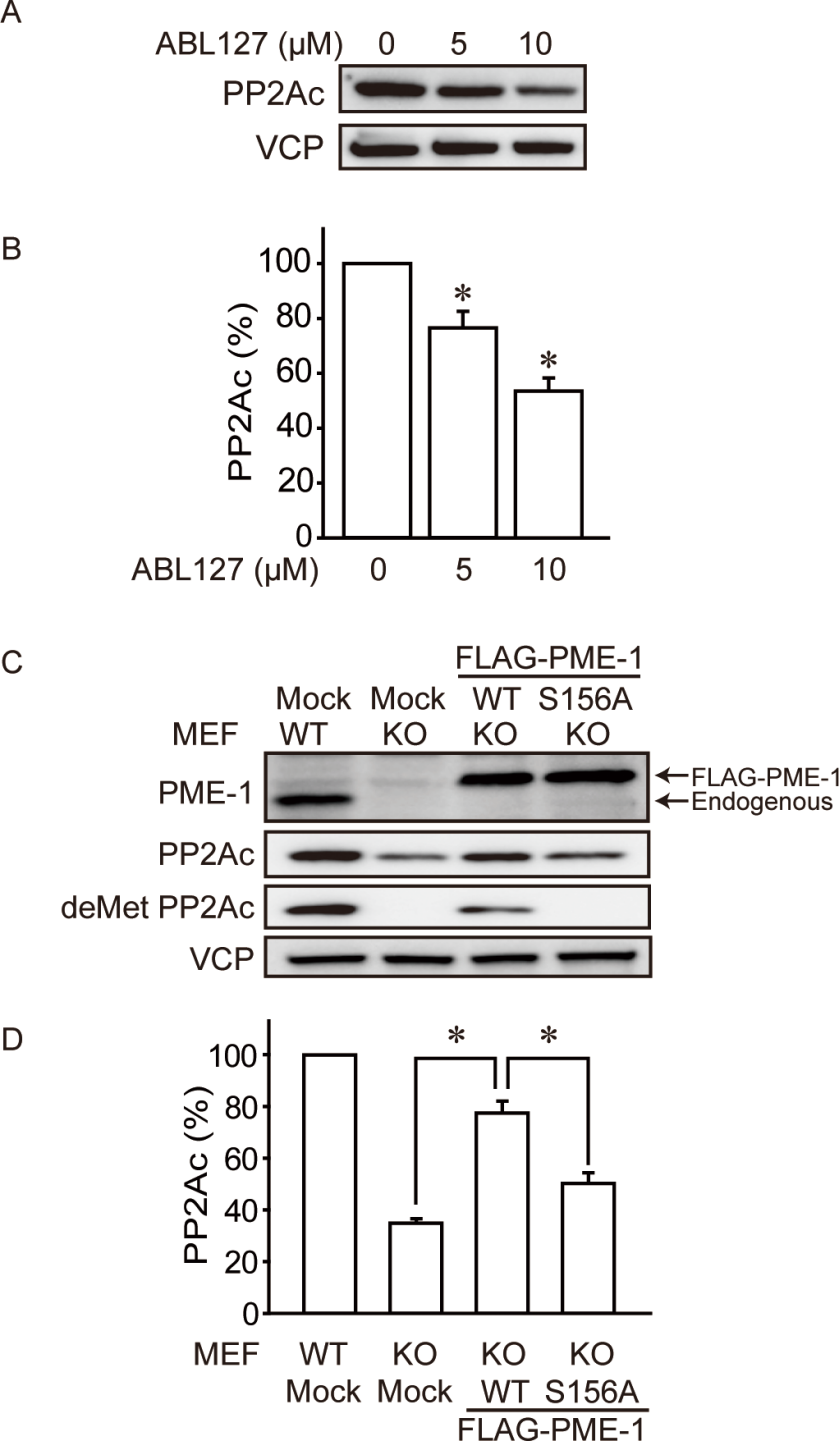
Methyl-esterase activity of PME-1 is required to maintain levels of PP2Ac. (A) PME-1 inhibitor decreases PP2Ac protein levels. WT MEFs were treated with ABL127 (5 or 10 μM) for 48 hr and levels of proteins were determined by immunoblotting. Representative images (A) and quantitative data (B) from 4 independent experiments are shown. *: *P*<0.05 vs. ABL127 untreated. (C-D) Methyl esterase activity of PME-1 is required to maintain PP2Ac levels. PME-1 KO MEFs were expressed FLAG-PME-1 WT or S156A (methyl esterase dead), and levels of proteins were determined by immunoblotting. Empty vector was used as mock. Representative images (C) and quantitative data (D) from 3 independent experiments are shown. *: *P*<0.05 vs. FLAG-PME-1 WT expressed KO MEFs.

We inhibited endogenous PME-1 in WT MEFs with a PME-1 selective chemical inhibitor ABL127 [21]. The effect of ABL127 was confirmed by detecting increased methylation levels of PP2Ac, but not protein level of PME-1 in cells with inhibited PME-1 (S3 Fig). ABL127 treatment for 48 hr suppressed protein levels of PP2Ac in a dose-dependent manner (Fig 5A and 5B). These results suggested to us that the methyl-esterase activity of PME-1 was required to maintain the levels of PP2Ac protein in living cells.

To confirm this point, we performed rescue experiment with WT and mutant PME-1. Substitution of Ser156Ala in PME-1 removes the essential active site nucleophile and abolishes the methyl esterase activity. However, this mutated PME-1 keeps the ability to bind the PP2Ac active site [14]. We confirmed this observation by immunoprecipitation that yielded about 2.5 times more PP2Ac bound to PME-1 S156A compared to PME-1 WT (S4 Fig). PME-1 KO MEFs were transfected to express either FLAG-PME-1 WT or S156A mutant. PME-1 KO MEFs infected with mock virus (empty FLAG vector) expressed 35.0 ± 2.5% of PP2Ac protein compared with WT MEFs (Fig 5C and 5D). Transfection with PME-1 WT and S156A mutant increased PP2Ac protein levels to 77.5 ± 7.8% and 50.3 ± 6.9%, respectively. Although S156A slightly rescued PP2Ac level, these data indicate that methyl-esterase activity of PME-1 is important to maintain levels of PP2Ac.

### PME-1 Knockout Suppresses Cell Proliferation

**Fig 6:**
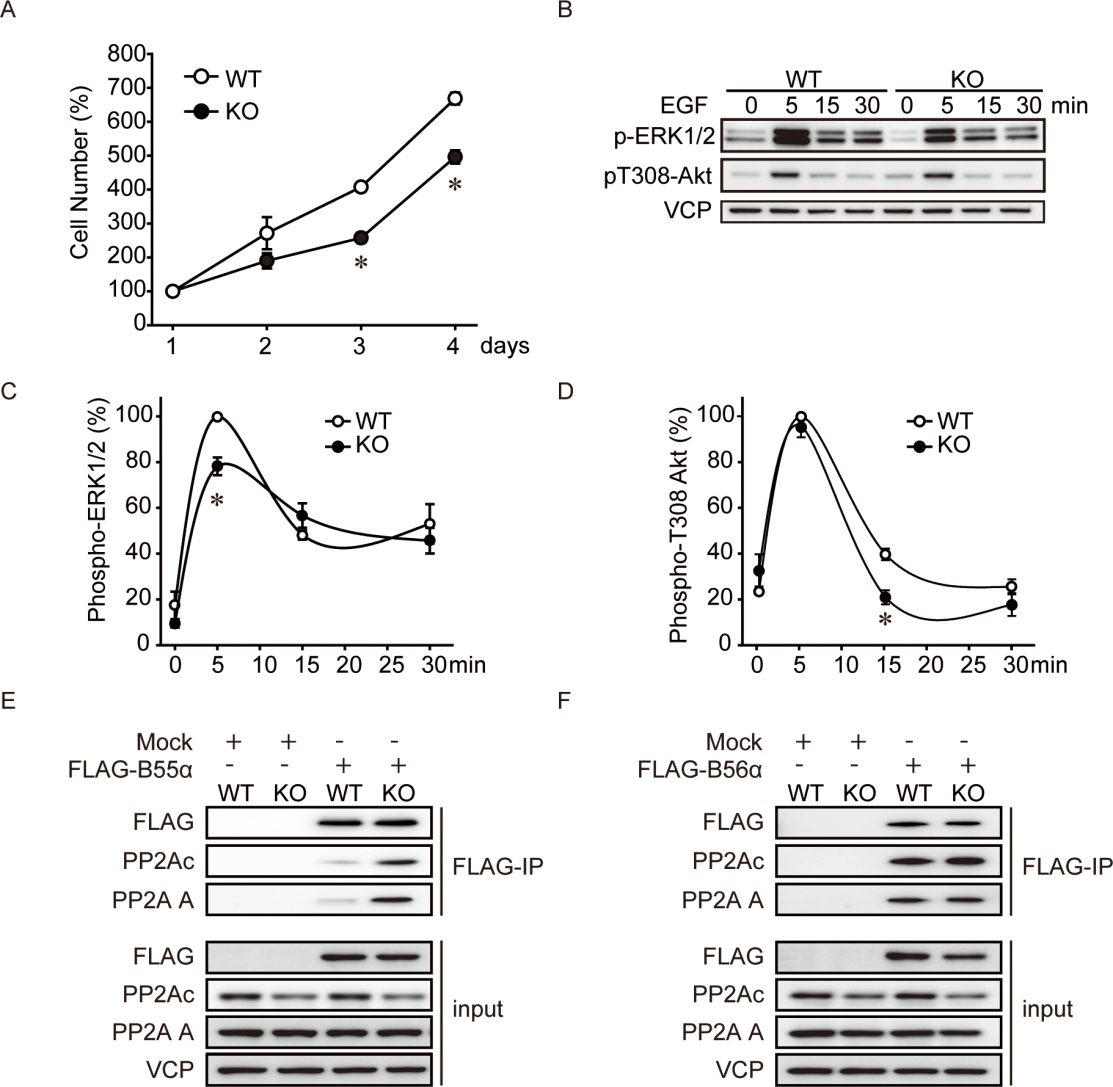
PME-1 knockout suppresses cell proliferation. (A) Loss of PME-1 suppresses cell proliferation. Cell proliferation of wild type (WT) and PME-1 KO (KO) MEFs were determined by Cell Counting Kit-8. N = 4 *: *P*<0.05 vs. WT. (B-D) Effects of PME-1 KO on ERK1/2 and Akt phosphorylation. WT and KO MEFs were stimulated with EGF (50 ng/ml) for indicated time periods and ERK1/2 and Akt phosphorylation was determined by immunoblotting. Representative images (B) from 3 independent experiments, and quantitative data for phospho-ERK1/2 (C) and phosphoT308 Akt (D) are shown. *: *P*<0.05 vs. WT. (E-F) Loss of PME-1 enhances the association of PP2A AB55αC complex. FLAG-B55α or B56α were transiently expressed in WT and PME-1 KO MEFs, and immunoprecipitated with FLAG-M2 beads. Empty vector was used as mock. PP2Ac and A subunit association was detected by immunoblotting. Representative images from 3 independent experiments were shown.

PME-1 knockdown by RNAi in tumor cells has been reported to suppress cell growth by PP2A activation—ERK1/2 dephosphorylation cascade [15, 18]. Because PP2A activity was reduced in PME-1 KO MEFs (Fig 1A and 1B), we analyzed effects of PME-1 KO on cell proliferation. Interestingly, our analysis showed reduced cell proliferation of PME-1 KO MEFs compared to WT MEFs (Fig 6A). To study the effect of PME-1 KO on ERK1/2 and Akt phosphorylation, WT and PME-1 KO MEFs were cultured in serum-free medium for overnight and stimulated with EGF (Fig 6B–6D). EGF-induced transient phosphorylation of ERK1/2 and sustained Akt Thr308 phosphorylation were slightly suppressed in PME-1 KO MEFs. Total protein levels of ERK1/2 and Akt were not different between WT and KO MEFs (S5 Fig). We hypothesized that the amount of PP2A heterotrimer targeting ERK1/2 and Akt signaling is increased in PME-1 KO MEFs. Consistent with this idea, we observed increased association of PP2Ac and A subunit with B55α subunit in PME-1 KO MEFs (Fig 6E). Therefore, the loss of PME-1 suppressed cell growth and activation of the ERK1/2 and Akt signaling pathway by increased number of PP2A-B55α complex known to target these 2 substrates. On the other hand, the association of PP2Ac with B56α subunit was not significantly different between WT and KO MEFs (Fig 6F), suggesting the relatively specific effects on PP2Ac-B55α complex.

## Discussion

**Fig 7:**
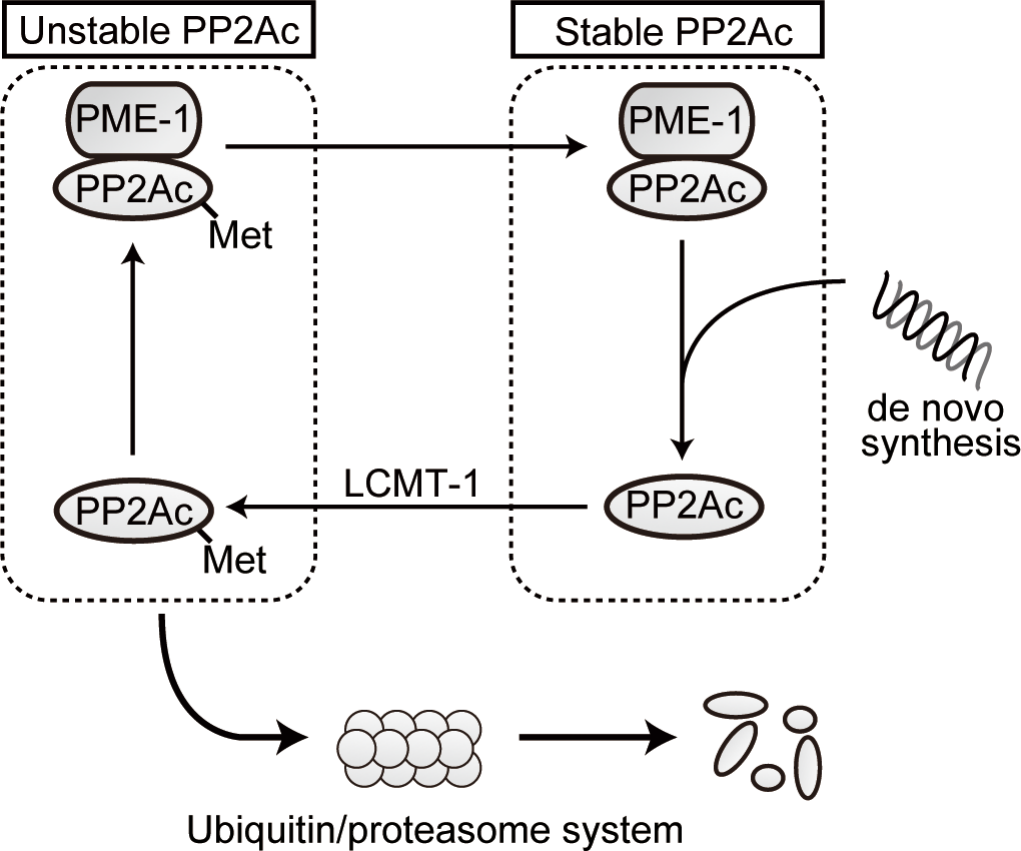
Proposed working hypothesis. Proposed working hypothesis for the regulatory mechanism for PP2A protein level by PME-1. See the Discussion for details.

In this report, we discovered that KO of PME-1 in primary MEFs reduced protein levels of PP2Ac, indicating a role for PME-1 in maintaining cellular levels of PP2Ac. Together with previous observations, we propose a hypothesis for how PP2Ac protein level is regulated by reversible methylation (Fig 7). Lack of PME-1 led to the almost complete methylation of PP2Ac, a change in the holoenzyme complement increasing the levels of PP2A-B55α trimers but most importantly to a substantial decrease of PP2Ac levels by increased poly-ubiquitination/degradation. Disturbance of the methyltransferase-methylesterase balance thus seem to interfere with proper holoenzyme biogenesis generating unstable methylated PP2Ac that is susceptible to poly-ubiquitination/degradation. In undisturbed cells, PME-1 binds to methylated PP2Ac and inhibits its activity but this association is not enough to protect completely PP2Ac from degradation as the methyl-esterase activity is important to stabilize PP2Ac. In the cells which lost PME-1, almost all PP2A is methylated and is prone to enhanced degradation by the ubiquitin/proteasome system.

The PP2Ac expression and activity are strictly regulated to balance phosphorylation and dephosphorylation of signaling pathways. As shown in S2 Fig, exogenous PP2Ac level is different between WT and KO cells 24 hr after doxycycline (dox) treatment, presumably by enhanced degradation in KO cells. However, when cells were treated with dox for longer time (64 hr), exogenous PP2Ac level become same between WT and KO cells (Fig 4A), suggesting the upper limit of exogenous PP2Ac protein. Consistent with this, ectopically expressed PP2Ac is restricted by an undefined autoregulatory feedback mechanism that maintains PP2A at constant levels [24]. These data suggest that there is PME-1 independent mechanism(s) to limit upper level of PP2Ac protein. Meanwhile, the range of PP2Ac protein expressions below the upper limit seems to be relatively wide-ranged, as long as whole cell PP2A activity is sufficient for cell viability. Although there is not a solid system to measure PP2A activity at present, we showed decreased PP2A activity by two different methods. We observed about 30% decrease in phosphatase activity in whole cell PP2A activity assay and about 50% decrease in PP2A activity by immunoprecipitation based activity assay (Fig 1A and 1B). It is possible that PP4 and PP6 contribute to the phosphatase activity in whole cell PP2A activity assay. Consistent with our data, previous work using PME-1 KO embryo tissues showed decreased PP2A activity [19].

PME-1 directly binds to the active site of PP2Ac and inhibits PP2A, methyl-esterase activity independent manner [14]. In our report, chemical inhibition and rescue experiments that reverse the effects of PME-1 deletion provide evidence that methyl-esterase activity of PME-1 is important for protection of PP2Ac from degradation. Immunoprecipitation using PME-1 WT and S156A showed more PME-1 S156A bound to PP2Ac compared to WT as previously shown [18], suggesting association of PME-1 is not sufficient to protect PP2Ac, even though activity dead PME-1 can rescue partially PP2Ac from degradation. These data indicate that the methylation state of Leu309 in PP2Ac is one of the important factors for PP2Ac stability. Methylation of PP2Ac affects interactions with the other proteins, such as α4 and B55/B56 subunits [11, 12, 25], thereby it may play a role in E3 ligase recognition.

The higher level of PP2Ac associated with the inactive PME-1 mutant than with WT suggests that demethylation of PP2Ac leads to dissociation of PP2Ac/PME-1 complex. This is consistent with product release from enzyme following catalysis (hydrolysis). How is PP2Ac kept inactive and protected from ubiquitination after dissociation of PME-1? One possible mechanism is the association with α4, which previously was reported to stabilize PP2Ac in an inactive form [26]. The α4 protein (Tap42 in yeast) is an evolutionally conserved essential factor which directly binds with PP2Ac without A subunit [27]. Recent evidence demonstrates that α4 has an ubiquitin-interacting motif and can prevent PP2Ac ubiquitin/proteasomal degradation [26, 28, 29]. In our system, the levels of α4 protein were not different between WT and KO MEFs nor did the levels of α4 protein associated with PP2Ac change (S6A and S6B Fig). Thus, decreased PP2Ac protein levels in PME-1 knockout were unlikely caused by a reduction in α4 levels. Alternatively, PP2Ac also could be stabilized by PP2A inhibitors or other post-translational modifications besides methylation of Leu309. PP2Ac is inactivated by association with inhibitors, such as SET and CIP2A, or by C-terminal Tyr307 phosphorylation [17, 30–32]. Mutation of Tyr307 of PP2Ac was shown to influence the association with α4 [25], suggesting the possibility that this modification influences PP2Ac stability via binding of α4. Transient suppression of SET or CIP2A did not reduce PP2Ac protein levels [17, 33], but knockout or stable knockdown systems should be used to clarify this point.

Decreased PP2Ac protein levels were not reported in previous study with PME-1 KD [15, 18]. We observed that PME-1 KD decreased PP2Ac protein in A549 cells but not in 293 and HT29 cells (S1 Fig). Therefore, the effect of PME-1 loss on PP2Ac protein levels is dependent on the cell types, and further study is necessary to clarify the mechanism behind this difference. Differences in the effect of PME-1 loss also produced different effects on growth factor signaling pathways. We observed that PME-1 KO had only minor effects on ERK1/2 and Akt phosphorylation (Fig 6B–6D), while previous works showed that suppression of PME-1 strongly inhibited them [15, 18]. Even though PP2A activity is decreased in PME-1 KO MEFs, phosphorylation levels of ERK1/2 were suppressed suggesting an increase of the specific B subunit driven activity. Indeed the methylation status of PP2Ac is known to affect the composition of PP2A heterotrimers. Therefore, loss of PME-1 may increase the pools of ABC trimers targeting PP2Ac to ERK1/2 and Akt at the expense of AC dimer and other ABC trimers. Consistent with this idea, we observed increased association of PP2Ac with B55α subunit in PME-1 KO MEFs (Fig 6E). PP2A complex with B55α has been reported to downregulate ERK1/2 signaling by inhibiting upstream kinase MAP3K2 [34], and Akt signaling by direct inhibition [35], suggesting increased number of PP2A-B55α complex leads to decreased ERK1/2 and Akt activity.

Loss of PME-1 specifically suppressed PP2Ac levels without a parallel decrease in the A subunit (Fig 2A–2C). Most of PP2Ac has been thought to exist as a complex with scaffolding A subunit, AC core dimers or ABC trimers [8, 36, 37]. However, accumulating evidence shows the lack of strict correspondence of A subunit and PP2Ac levels under certain circumstances. Knockdown of PP2Ac by siRNA in T cells did not cause a corresponding reduction in A subunit [38]. Conversely, >60% decrease in A subunit by shRNA reduced PP2Ac by only 30% in PC6-3 cells [39]. Moreover, large variation in the levels of A subunit, but not in PP2Ac, were observed in human gliomas [40]. Therefore, the protein levels of PP2Ac and A subunit might be separately regulated. In yeast, the PME-1 homologue plays a role in an early step of PP2A biogenesis, namely as a mechanism to surveil and restore the correct order of PP2A biogenesis [9]. Although PME-1 can associate with PP2A AC dimer and ABC trimer [41], it is possible that PME-1 primarily exerts its effects on monomeric PP2Ac.

Collectively, our results provide a deeper understanding for the mechanism and importance of PME-1 in control of PP2A.

## Supporting Information

S1 Fig Effect of PME-1 loss on PP2Ac protein levels is dependent on cell types. Effect of PME-1 loss on PP2Ac protein levels is dependent on cell types. A549 (A), 293 (B), and HT29 (C) cells were stable expressed non-targeting shRNA (shNT) or shRNA targeting PME-1 (shPME-1). Levels of proteins were determined by immunoblotting. Representative images from 3 independent experiments and quantitative data are shown. *: P<0.05 vs. shNT.
(TIF)

S2 Fig Level of FLAG-PP2Ac protein after short time treatments with dox. (A) Wild type (WT) and PME-1 knockout (KO) MEFs were treated with doxycycline (dox) for 12 or 24 hr, and FLAG-PP2Ac was expressed by TetOn system. Level of FLAG-PP2Ac was detected by immunoblotting. Representative images from 3 independent experiments were shown. (B) PME-1 KO MEFs were treated with dox for 24 hr, and FLAG-PP2Ac WT or K41R were expressed by TetOn system. Level of FLAG-PP2Ac was detected by immunoblotting. Representative images from 3 independent experiments were shown.
(TIF)

S3 Fig ABL127 enhanced methylation levels of PP2Ac. WT MEFs were treated with or without ABL127 (5 μM) for 48 h. PP2Ac methylation levels and PME-1 protein levels were detected by immunoblotting with anti-demethylated PP2Ac and anti-PME-1 antibody, respectively. Representative images from 3 independent experiments are shown.
(TIF)

S4 Fig Methyl-esterase activity of PME-1 is not necessary for association with PP2Ac. FLAG-PME-1 WT and S156A mutant were transiently expressed in 293T cells, and immunoprecipitated with FLAG-M2 beads. PP2Ac association was detected by immunoblotting. Representative images from 2 independent experiments were shown.
(TIF)

S5 Fig Levels of ERK1/2 and Akt protein in WT and KO MEFs. Levels of proteins in WT and PME-1 KO MEFs were determined by immunoblotting and representative images and quantitative data for ERK1/2 (A), and Akt (B) from 3 independent experiments are shown.
(TIF)

S6 Fig PME-1 knockout do not change α4 protein expression and PP2Ac-α4 interaction. (A) Levels of proteins in wild type (WT) and PME-1 KO (KO) MEFs were determined by immunoblotting and representative images and quantitative data for α4 from 3 independent experiments are shown. (B) PP2Ac was immunoprecipitated with anti-PP2Ac from WT and PME-1 KO MEFs. α4 association was detected by immunoblotting. Representative images from 3 independent experiments were shown.
(TIF)
