# Supplementary figures and images for: Protein Phosphatase Methyl-Esterase PME-1 Protects Protein Phosphatase 2A from Ubiquitin/Proteasome Degradation
Source: PLoS One. 2015 Dec 17;10(12):e0145226. doi: 10.1371/journal.pone.0145226 (PMC4683032; doi:10.1371/journal.pone.0145226)

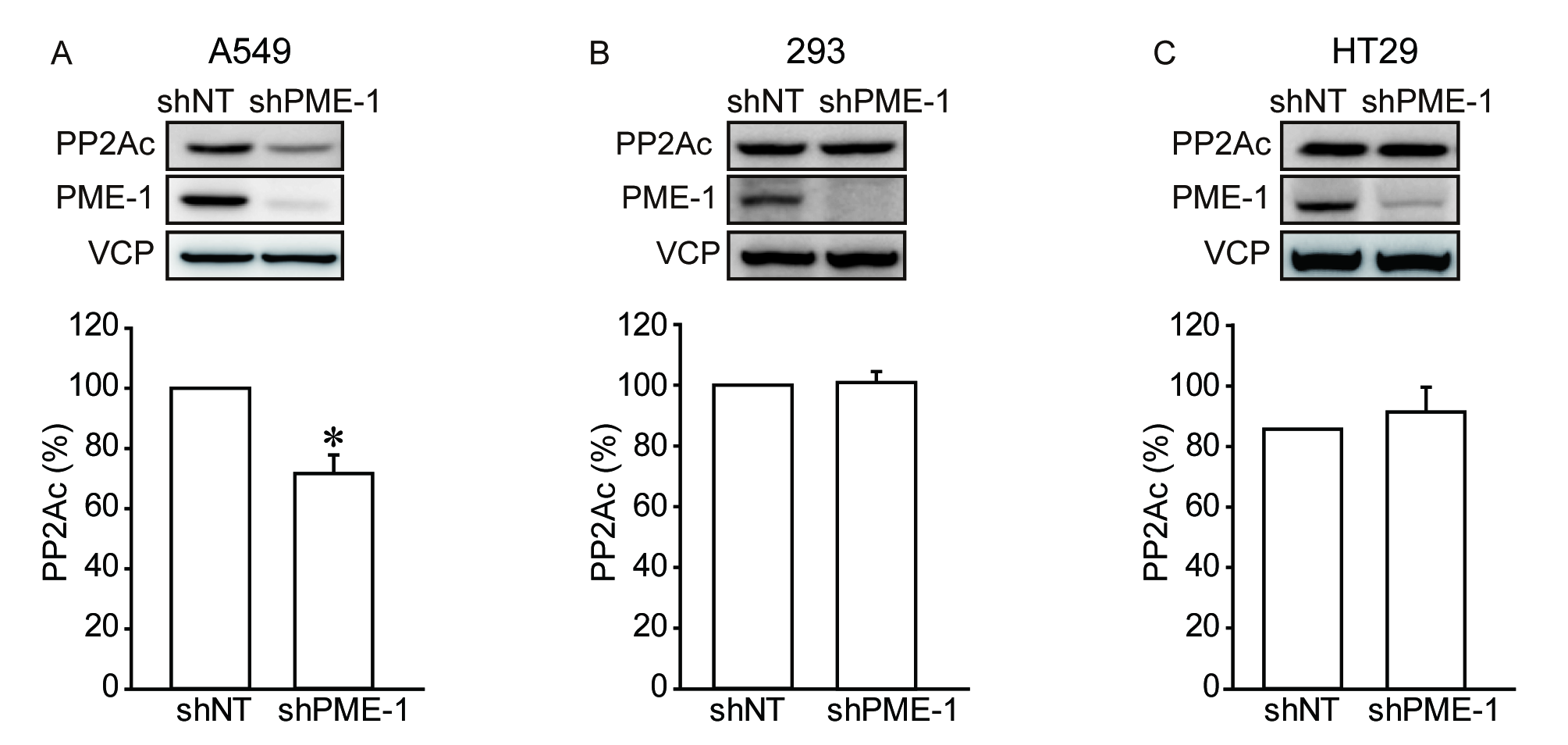

Supplement: S1 Fig — Effect of PME-1 loss on PP2Ac protein levels is dependent on cell types. A549 (A), 293 (B), and HT29 (C) cells were stable expressed non-targeting shRNA (shNT) or shRNA targeting PME-1 (shPME-1). Levels of proteins were determined by immunoblotting. Representative images from 3 independent experiments and quantitative data are shown. *: P<0.05 vs. shNT. (TIF) [file pone.0145226.s001.tif]

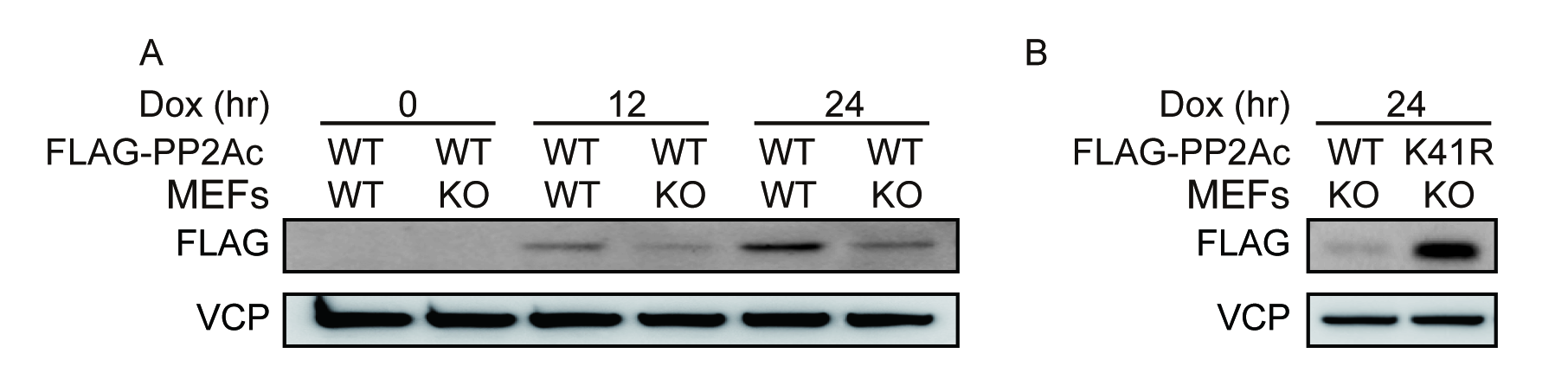

Supplement: S2 Fig — (A) Wild type (WT) and PME-1 knockout (KO) MEFs were treated with doxycycline (dox) for 12 or 24 hr, and FLAG-PP2Ac was expressed by TetOn system. Level of FLAG-PP2Ac was detected by immunoblotting. Representative images from 3 independent experiments were shown. (B) PME-1 KO MEFs were treated with dox for 24 hr, and FLAG-PP2Ac WT or K41R were expressed by TetOn system. Level of FLAG-PP2Ac was detected by immunoblotting. Representative images from 3 independent experiments were shown. (TIF) [file pone.0145226.s002.tif]

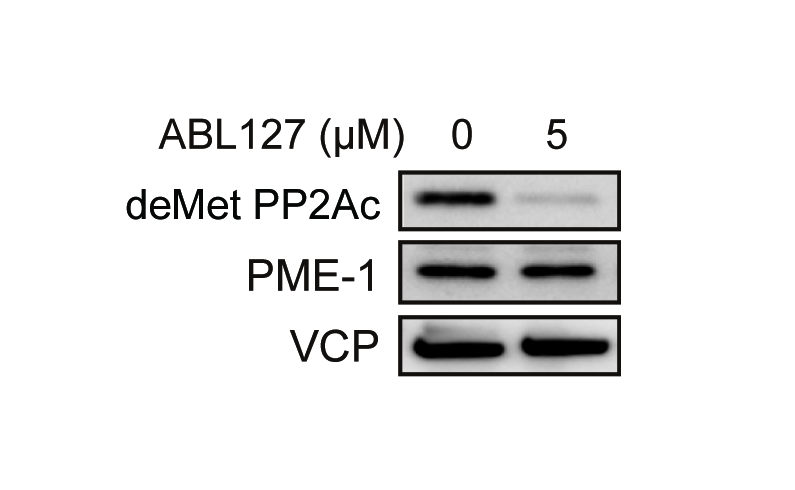

Supplement: S3 Fig — WT MEFs were treated with or without ABL127 (5 μM) for 48 h. PP2Ac methylation levels and PME-1 protein levels were detected by immunoblotting with anti-demethylated PP2Ac and anti-PME-1 antibody, respectively. Representative images from 3 independent experiments are shown. (TIF) [file pone.0145226.s003.tif]

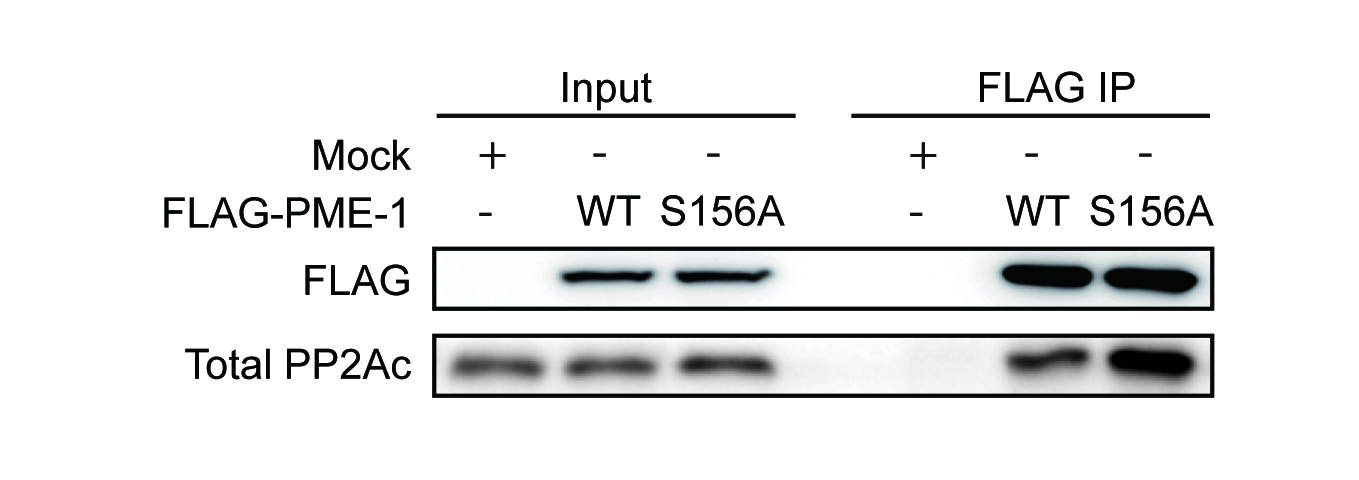

Supplement: S4 Fig — FLAG-PME-1 WT and S156A mutant were transiently expressed in 293T cells, and immunoprecipitated with FLAG-M2 beads. PP2Ac association was detected by immunoblotting. Representative images from 2 independent experiments were shown. (TIF) [file pone.0145226.s004.tif]

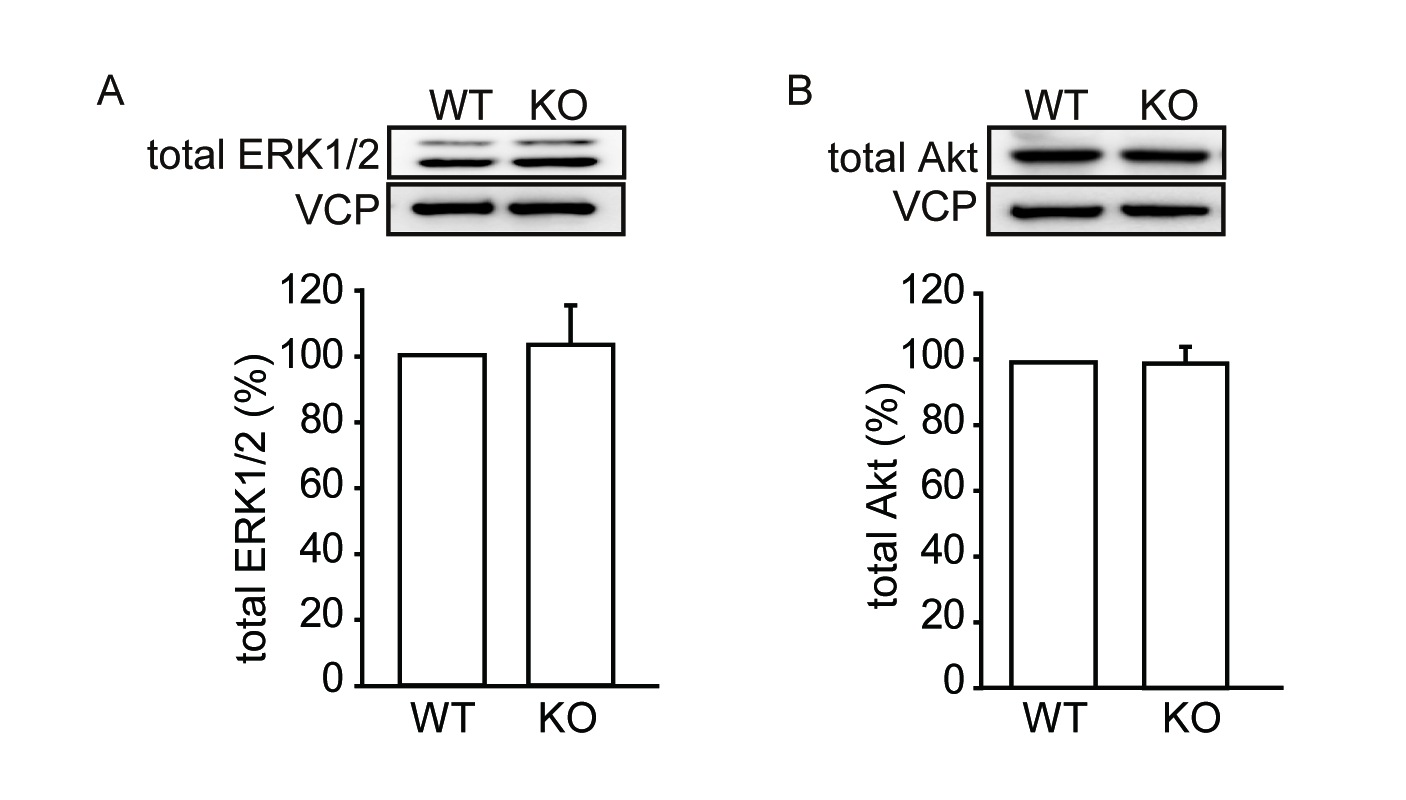

Supplement: S5 Fig — Levels of proteins in WT and PME-1 KO MEFs were determined by immunoblotting and representative images and quantitative data for ERK1/2 (A), and Akt (B) from 3 independent experiments are shown. (TIF) [file pone.0145226.s005.tif]

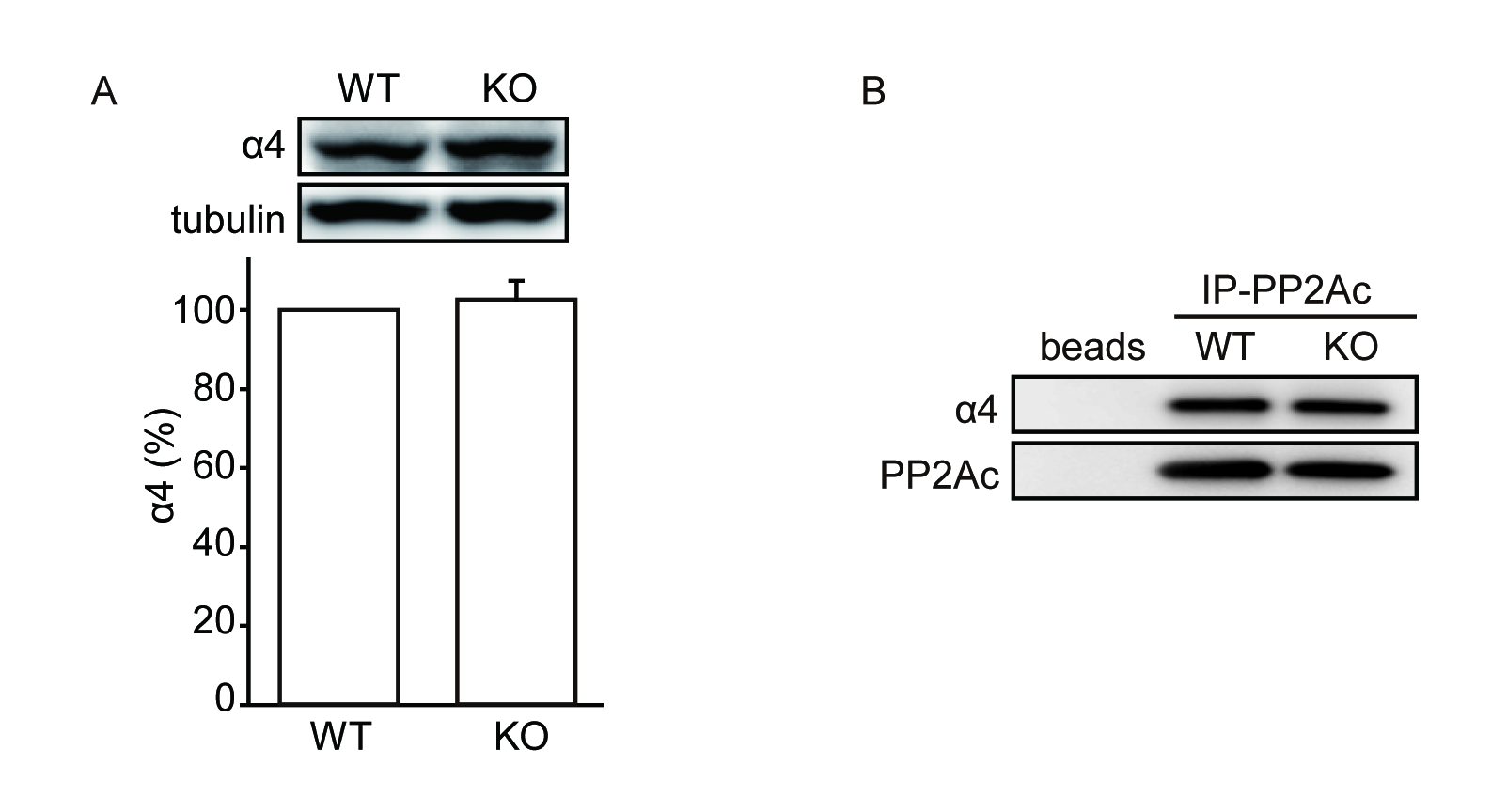

Supplement: S6 Fig — (A) Levels of proteins in wild type (WT) and PME-1 KO (KO) MEFs were determined by immunoblotting and representative images and quantitative data for α4 from 3 independent experiments are shown. (B) PP2Ac was immunoprecipitated with anti-PP2Ac from WT and PME-1 KO MEFs. α4 association was detected by immunoblotting. Representative images from 3 independent experiments were shown. (TIF) [file pone.0145226.s006.tif]
